# *Tofu*: a fast, versatile and user-friendly image processing toolkit for computed tomography

**DOI:** 10.1107/S160057752200282X

**Published:** 2022-04-04

**Authors:** Tomáš Faragó, Sergey Gasilov, Iain Emslie, Marcus Zuber, Lukas Helfen, Matthias Vogelgesang, Tilo Baumbach

**Affiliations:** a Institute for Photon Science and Synchrotron Radiation, Karlsruhe Institute of Technology (KIT), Herrmann-von-Helmholtz-Platz 1, 76344 Eggenstein-Leopoldshafen, Germany; b Canadian Light Source, 44 Innovation Blvd, Saskatoon, Canada S7N 2V3; c Laboratory for Applications of Synchrotron Radiation, Karlsruhe Institute of Technology, Kaiserstrasse 12, 76131 Karlsruhe, Germany; d Institut Laue-Langevin, 71 Avenue des Martyrs, CS 20156, 38042 Grenoble Cedex 9, France; e Institute for Data Processing and Electronics, Karlsruhe Institute of Technology, Hermann-von-Helmholtz-Platz 1, 76344 Eggenstein-Leopoldshafen, Germany

**Keywords:** tomography, laminography, parallel beam, cone beam, 3D reconstruction, phase retrieval, artifact removal, GPU computing, user interface, batch processing, visual programming

## Abstract

*Tofu* is a toolkit for processing large amounts of images and for tomographic reconstruction. Complex image processing tasks are organized as workflows of individual processing steps. The toolkit is able to reconstruct parallel and cone beam as well as tomographic and laminographic geometries. Many pre- and post-processing algorithms needed for high-quality 3D reconstruction are available, *e.g.* phase retrieval, ring removal and de-noising. *Tofu* is optimized for stand-alone GPU workstations on which it achieves reconstruction speed comparable with costly CPU clusters. It automatically utilizes all GPUs in the system and generates 3D reconstruction code with minimal number of instructions given the input geometry (parallel/cone beam, tomography/laminography), hence yielding optimal run-time performance. In order to improve accessibility for researchers with no previous knowledge of programming, *tofu* contains graphical user interfaces for both optimization of 3D reconstruction parameters and batch processing of data with pre-configured workflows for typical computed tomography reconstruction. The toolkit is open source and extensive documentation is available for both end-users and developers. Thanks to the mentioned features, *tofu* is suitable for both expert users with specialized image processing needs (*e.g.* when dealing with data from custom-built computed tomography scanners) and for application-specific end-users who just need to reconstruct their data on off-the-shelf hardware.

## Abstract 2

The versatile and user-friendly image processing toolkit *tofu*, optimized for 3D reconstruction of parallel beam, cone beam, tomography and laminography data, is presented.

## 1. Introduction

X-ray microtomography (µCT) is an invaluable non-invasive imaging technique for examining the internal structure of objects and organisms. Depending on a particular geometry [parallel or cone beam, tomography or laminography (Helfen *et al.*, 2011 ▸)] and source (X-ray, neutron,…), 3D reconstruction workflows may contain a lot of pre- and post-processing steps, including image normalization (Van Nieuwenhove *et al.*, 2015 ▸; Jailin *et al.*, 2017 ▸), phase retrieval (Paganin *et al.*, 2002 ▸; Moosmann *et al.*, 2011 ▸), de-noising (Buades *et al.*, 2005 ▸) and artifact removal (Hsieh *et al.*, 2000 ▸; Münch *et al.*, 2009 ▸; Vo *et al.*, 2018 ▸; Croton *et al.*, 2019 ▸). The final, high-quality workflow may contain a lot of image processing algorithms and parameters which need to be adjusted for each data set separately in an iterative manner and this might become overwhelmingly difficult for inexperienced users. Moreover, such workflows may be computationally demanding, which is not an issue for large facilities because they typically have access to computer clusters, but it becomes a problem for smaller laboratories that do not have access to such equipment where scientists must process the data on their own, often on relatively inexpensive hardware.

Computational speed is even more important if we consider the technological progress in imaging instrumentation. Thanks to continuous new developments in radiation sources (Raimondi, 2016 ▸; Schroer *et al.*, 2018 ▸), imaging detectors (Mokso *et al.*, 2017 ▸), and automation (Vogelgesang *et al.*, 2016 ▸; Marone *et al.*, 2017 ▸; Hashem *et al.*, 2021 ▸), the throughput of imaging systems is increasing. It is common that during one experimental day at a synchrotron imaging station researchers collect hundreds of data sets amounting to several terabytes of data. Even in a laboratory environment, modern µCT scanners enable data acquisition at the rate of just several tens of seconds per scan. This is highly demanded in order to capture transient processes and conduct *in situ* and *operando* studies. In this case, as well as when studying live animals under anesthesia, being able to preview and evaluate the image quality very quickly is extremely important for making a decision about repeating the scan or proceeding to the next specimen or operating point.

Based on the above observations, reconstruction software should be fast, versatile, user-friendly and scalable (capable of processing large volumes of data on inexpensive, off-the-shelf hardware). Fast reconstruction immediately after the data acquisition is needed to quickly assess the measurement results. A selection of common image pre- and post-processing operations, alongside suppression of typical artifacts, must be available in order to create flexible data processing workflows. It is beneficial to avoid using several different software tools since this always slows down the reconstruction process and creates unnecessary copies of data. In addition, a simple user interface is necessary for researchers without prior experience in scripting and programming. Scalability is very important for those who do not have access to a computer cluster but still need to process large amounts of data in their home laboratories.

Several open-source tomographic reconstruction tools are available. *Astra toolbox* (van Aarle *et al.*, 2016 ▸) offers multiple algorithms for both parallel and cone-beam computed tomography (CT) geometries; *PyHST2* (Mirone *et al.*, 2014 ▸) offers excellent run-time performance; *Tomopy* (Gürsoy *et al.*, 2014 ▸), *Savu* (Atwood *et al.*, 2015 ▸) and the *Syrmep Tomo Project* (Brun *et al.*, 2017 ▸) provide complex capabilities by auxiliary pre- and post-processing algorithms and interfaces to other 3D reconstruction tools. They provide command line interfaces (CLI), graphical user interfaces (GUI), or both.

In contrast to the flexibility of open source projects, reconstruction software supplied with laboratory scanners and commercially available reconstruction software like *Octopus* (Vlassenbroeck *et al.*, 2006 ▸) or *VG Studio Max* (https://www.volumegraphics.com/en/products/vgsm/ct-reconstruction-data-quality-analysis.html) are normally a black box, albeit with a very nice GUI, which reconstructs data following a predefined workflow. However, most of these systems cannot be modified to incorporate external algorithms into the reconstruction chain, for instance, trying a new phase-retrieval method. At the same time, there are more and more communications on custom-build laboratory systems for X-ray µCT [see Müller *et al.* (2017 ▸) and references therein, and Polyakov *et al.* (2017 ▸)]. Availability of user-friendly, easily extensible, and open-source software which can be used for phase-retrieval and reconstruction of cone-beam data can certainly facilitate new developments.

Here, we present *tofu*, a Python software package for general image processing, but with special emphasis on tomographic reconstruction that supports parallel, cone beam, tomographic and laminographic geometries. It is user-friendly without compromising processing speed and flexibility provided by the *ufo framework* (Vogelgesang *et al.*, 2012 ▸) back-end.

Unlike the existing open source tools, *tofu* connects the image processing algorithms into a workflow on the *ufo framework* (OpenCL) layer. Once the data is loaded, it stays in the GPU memory as it passes through the workflow. Hence, the processing time is significantly reduced as there is no need to download intermediate results to the main memory in order to pass them to the the subsequent stages of the workflow. *Tofu* is further equipped with versatile graphical user interfaces. *Tofu flow* is a GUI for visual programming of image processing workflows. Thanks to the fast execution, one can vary the parameters and observe changes in the output *interactively*. Once the optimal combination of algorithms and parameters is found, *tofu ez* can be used to automatically reconstruct multiple tomographic data sets.

In addition to the GUIs, there is a multitude of command line interface (CLI) sub-commands for experienced users, which can be embedded into scripts and other programs.

Three-dimensional (3D) reconstruction in *tofu* is flexible and supports complex scanning scenarios, such as helical CT. It includes algorithms for reduction of typical artifacts, *e.g.* rings, ‘zingers’ and noise. Several phase retrieval algorithms and many general image processing filters (*e.g.* pad, crop, blur) are available as well.

Scalability for 3D reconstruction is achieved by automatically splitting the data to all available GPUs in the system and by sub-dividing the final volume into several sub-volumes if needed. *Tofu* is open source with extensive documentation available online for both end-users and developers.

In the next section a brief description of the software structure is presented; the most important and frequently used algorithms are listed and the implementation of filtered back projection is described in more detail. Section 3 is dedicated to the user interfaces. Section 4 presents several application highlights, while Section 5 contains benchmarking results.

## 2. Ufo framework

*Tofu* is based on the open source *ufo framework* (Vogelgesang *et al.*, 2012 ▸), which (1) provides a large library of image processing algorithm implementations, (2) connects them into a workflow (a directed acyclic graph), and (3) executes these workflows on a broad range of computer systems. The framework is written in C in a cross-platform and binding-friendly way, so that it can be used on different operating systems (Linux, Mac, Windows) and accessed from different programming languages (*e.g.* Python). It uses OpenCL for hardware-agnostic parallelization which allows for efficient execution on both CPUs and GPUs from various vendors, including NVIDIA, AMD and Intel. From the user perspective, there is a detailed description on how to install the prerequisites and the software itself on different operating systems and processor architectures in the manual (https://ufo-core.readthedocs.io/en/latest/install/index.html). Moreover, there are various Docker images available for download on Docker Hub (https://hub.docker.com/r/tfarago/ufo-kit), which allows users to skip the installation step on Linux. The framework can read *raw*, *tif*, *hdf5* and *edf* file types and write *raw*, *tif*, *hdf5* and *jpg*. Table 1 ▸ summarizes the components of the software stack behind *tofu*.

Currently, there are over 90 image processing algorithms implemented in *ufo-filters*; here we will shortly describe the ones we implemented for obtaining high-quality 3D reconstruction.

**2D Phase retrieval** algorithms for near-field Fresnel diffraction images (also known as propagation-based phase contrast images) have been implemented, including the transport-of-intensity method (Paganin *et al.*, 2002 ▸) and various contrast transfer function approaches (Moosmann *et al.*, 2011 ▸). Moreover, the combination of multiple object–detector distances is supported as well (Zabler *et al.*, 2005 ▸). For the case of a single object–detector distance, *x*- and *y*-direction distances can be specified separately, which is useful for processing images with non-symmetrical pixel size or various propagation distances, like in the case of Bragg magnifier imaging (Vagovič *et al.*, 2014 ▸).

**Figure 1:**
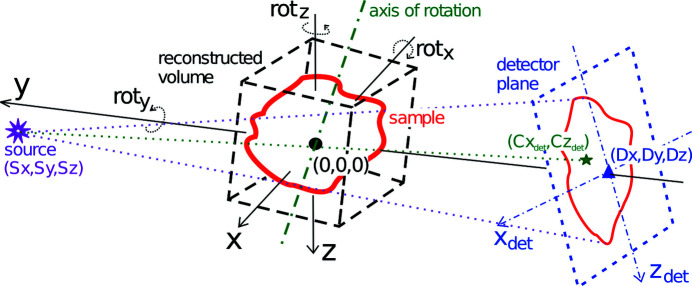
Reconstruction geometry. The center of origin is in the center of the reconstructed volume. Positions of the source and the detector are defined with respect to this point. Point ( 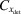 , 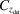 ) is the projection of the center of rotation on the detector plane. The detector, the rotation axis and the reconstructed volume can be arbitrarily oriented.

**3D reconstruction** is realized by the filtered back projection (FBP) algorithm for parallel beam and by the Feldkamp approach (Feldkamp *et al.*, 1984 ▸) for cone beam data. Rotations of the detector, the axis of rotation and the reconstructed volume are supported for the treatment of a static setup mis­alignment, the possibility to reconstruct laminography data and the ability to rotate the reconstructed volume without the need for post-reconstruction 3D rotation. In addition, the reconstruction parameters can be specified for every projection separately to permit dynamic changes in the setup. This enables complex reconstructions without the need of data pre-processing, *e.g.* helical CT and motion-compensated reconstruction in case of vibrations or systematic drift. Fig. 1 ▸ depicts the geometrical setup in more detail. Before the final reconstruction, it is often necessary to find the correct values of the reconstruction parameters, *e.g.* center of rotation, laminographic angle, *etc*. In order to help find these, the output of the algorithm is a 3D volume of horizontal slices where the third dimension does not need to be the vertical slice position. Instead, one can reconstruct the same horizontal slice with different values of a reconstruction parameter. Some metric, *e.g.* the standard deviation, can then be applied to such output to find the correct parameter value. Based on the specified geometry, an optimized back projection OpenCL kernel code with a minimal number of mathematical operations is generated at run-time, which leads to optimal reconstruction speed (*e.g.* coordinate transformations required for the tilted rotation axis in the case of laminography may be omitted in the case of tomography). On the top of that, only projection regions necessary for the specified reconstructed volume are read from the input data in order to minimize I/O.

**Ring artifact removal** is based on two algorithms, one for removing narrow and one for removing broad rings.

Narrow rings, often stemming from noise, are removed by suppressing stripes which are close to being vertical in a sinogram (a row in a sinogram represents a projection under a certain angle), implemented by filtering the 2D Fourier transform of the sinogram.

Broad rings, typical for scintillator defects, are filtered by locating the corresponding spots of extreme intensity in the projections by thresholding and region growing which yields a mask representing invalid pixels. Horizontal linear interpolation is then applied to replace erroneous intensity values.

The **Non-local means noise removal** algorithm has been shown to significantly improve the signal-to-noise ratio of filtered images while preserving fine details (Buades *et al.*, 2005 ▸). Such a filter is very desirable for the processing of low signal-to-noise ratio data, stemming from *e.g.* high-speed synchrotron experiments, high magnifications at lab sources, or neutron sources. Our current implementation supports the original algorithm and the faster version based on cumulative sums (Darbon *et al.*, 2008 ▸).

## 3. User interfaces

In this section, we will describe user interfaces (UIs) for working with the *ufo framework*. Our primary focus will be the two GUIs *tofu flow* and *tofu ez*, which enable user-friendly creation of image processing workflows and batch processing of tomographic data sets. We will also briefly describe the CLIs; Table 2 ▸ contains the complete list of the user interfaces.

With any of the interfaces, the 3D reconstruction produces quantitatively the following output:

(i) In the case of monochromatic absorption input data, the voxel values are unitless and correspond to Δ*x*μ, where Δ*x* is the pixel size of the detector and μ the linear attenuation coefficient.

(ii) In the case of phase retrieval applied on projections (and within the approximation limits of the respective retrieval algorithm), the voxels correspond to either the unitless phase shift −2πΔ*x*δ/λ or, in the case of the transport-of-intensity method and specification of the δ part of the refractive index, the Δ*x* in meters.

(iii) After ring removal, the results can no longer be quantitatively interpreted.

### 3.1. *Tofu* flow

**Figure 2:**
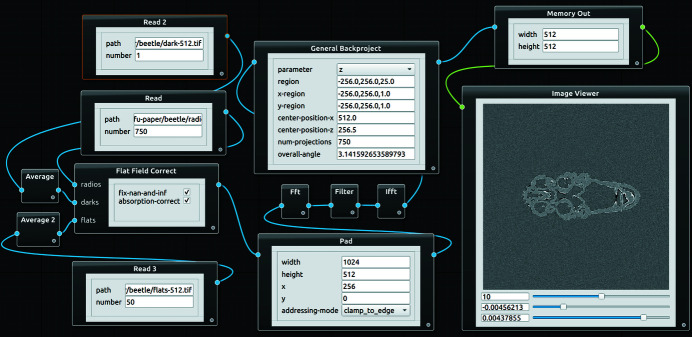
Main window of *tofu flow* showing tomographic reconstruction. Dark and flat field images are averaged and used to flat-field-correct the radiographs. The normalized images are padded in order to remove convolution outlier artifacts, Ram-Lak-filtered in the Fourier space, back projected and displayed.

*Tofu flow* is a GUI (Fig. 2 ▸) for visual composition of image processing workflows. Its main advantage is quick and flexible flow creation with embedded visualization of results. This combination makes the program *interactive*, which is very desirable when it comes to optimization of image processing parameters or education and training of people who are not yet familiar with tomographic image processing. It is written in Python 3 and PyQT5 (https://riverbankcomputing.com/software/pyqt); the flow scene is based on *qtpynodeeditor* (https://github.com/klauer/qtpynodeeditor).

Algorithms from the *ufo framework* in the flow scene are represented as graphical nodes. Several nodes can be combined into one composite node in order to remove clutter. Algorithm parameters can be set directly inside nodes using standard input widgets from the Qt (https://www.qt.io) library. Moreover, parameters can be linked between nodes, *i.e.* changing one node’s parameter automatically updates another one’s, which minimizes the amount of required manual adjustments. Flow direction is defined by node connections. Starting points are nodes representing a data source and do not have input connections (*e.g.* Read node). Typical processing nodes have inputs and an output; the flow ends in sink nodes which do not have an output. Apart from write sink node, which writes the results to a disk, there is also an Image Viewer node for quick visualization of 2D images or 3D volumes.

**Figure 3:**
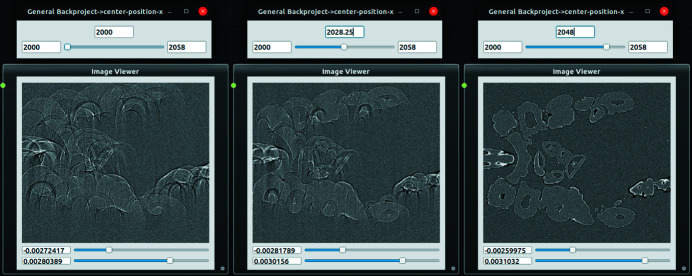
An example of interactive parameter adjustment in *tofu flow*. The user gradually changes the center of rotation value from 2000 to 2048 by dragging a slider and the updated slice is immediately shown.

Execution of a flow in the scene, including scheduling and utilization of all GPUs in the system, is by default left to *ufo-core*. In the case of the memory-demanding 3D reconstruction, *tofu flow* creates several batches and executes them in sequence if more data than what fits into the GPU memory needs to be reconstructed. In order to provide an interactive way of working with flows, node parameters can be adjusted by a slider. Once its value has changed, a flow execution is triggered and the result in the image viewer is updated, see Fig. 3 ▸.

### 3.2. Tofu ez

**Figure 4:**
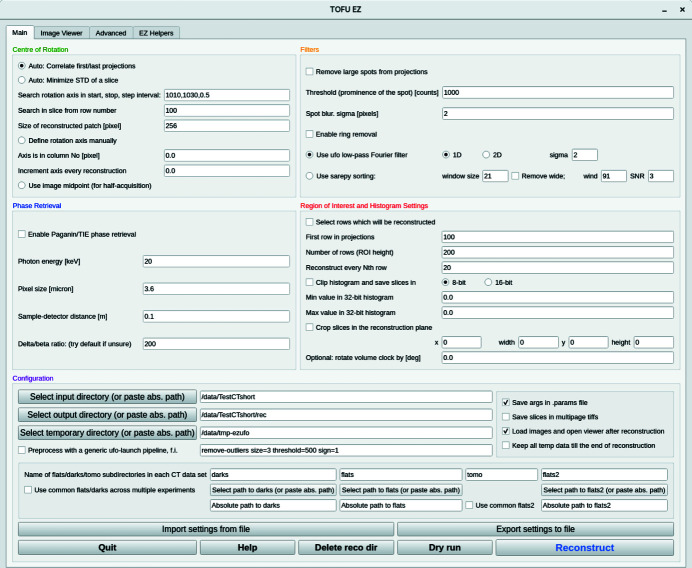
Main window of the *tofu ez* interface.

Writing custom scripts for batch processing of data allows one to tailor the reconstruction workflow perfectly for each particular case. However, not every research group can afford to have a person with a computer science background or an image processing specialist. To address this problem we have developed a user-friendly interface *tofu ez* which can be used to reconstruct data by scientists without substantial knowledge of the Linux command line or Python scripting skills. *Tofu ez* (Fig. 4 ▸) simplifies the usage of *ufo-launch* and *tofu* by exposing all important parameters in a PyQT5-based interface and automatically formatting a suitable list of commands depending on the user input. Typical applications of *tofu ez* include:

(i) Optimization of reconstruction parameters.

(ii) Single-click reconstruction of freshly acquired data during the experiment.

(iii) Horizontal and vertical stitching of adjacent CT volumes.

(iv) Batch processing of data after the experiment.

(v) Data reduction and preparation for further analysis and 3D visualization.

In order to start using *tofu ez* one prerequisite must be fulfilled: tomographic projections and auxiliary images required for intensity normalization (images acquired with sample moved out of the beam and detector background noise images, colloquially referred to as flats and darks) must be saved in separate directories as separate tif files or in a bigtiff container.

**Figure 5:**
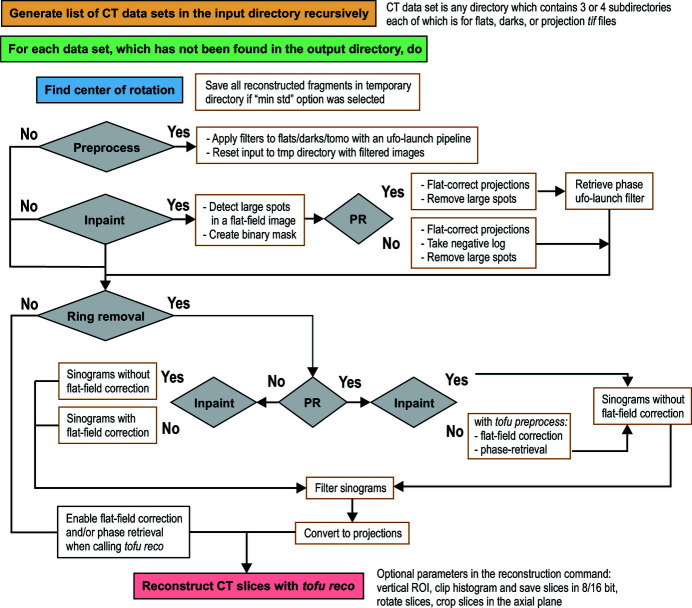
Block diagram of the *tofu ez* workflow generation. PR stands for phase retrieval; inpaint refers to an algorithm which removes large spots stemming from scintillator defects.

At the beginning of a reconstruction, *tofu ez* creates a list of paths to all valid CT directories in the input directory. The names of CT directories are compared with the directory tree in the output directory (the relative path to a CT set in the input directory is preserved when results are saved in the output directory). Those CT sets whose names are not yet in the output directory will be reconstructed. If requested, *tofu ez* will automatically estimate the center of rotation parameter, which is the only unknown variable in the input of the filtered backprojection algorithm in parallel beam geometry. This information is used during the second pass, when the program creates an array of *ufo-launch* and *tofu* commands according to defined parameters and then executes them sequentially. The commands can also be printed on the screen. Fig. 5 ▸ shows how *tofu ez* creates workflows in different situations. Metadata can be loaded from a configuration file and all parameters that could be used to re-run the reconstruction are saved automatically along with the reconstructed data.

Operations belonging to the following 12 categories can be chained together to form a workflow which can be applied to multiple data sets in the input directory automatically:

(i) Horizontal stitching of half acquisition mode data (see explanation in the paragraph below the list).

(ii) Pre-processing with arbitrary *ufo-launch* workflow; default option is the remove-outliers filter for the suppression of ‘zinger’ artifacts.

(iii) Removal of large spots which stem from defects in the scintillator crystal.

(iv) Flat-field correction including dynamic intensity normalization (Van Nieuwenhove *et al.*, 2015 ▸).

(v) Phase-retrieval.

(vi) Filtration of sinograms for the suppression of ring artifacts; fast Fourier transform based filter as well as a method which does not rely on the Fourier transform (Vo *et al.*, 2018 ▸) are available.

(vii) Automatic estimation of the position of the center of rotation; two different algorithms are available.

(viii) Tomographic reconstruction with *tofu reco*.

(ix) Crop output slices and rotate the object within the reconstructed slice.

(x) Clip the histogram and convert reconstructed values to either 8 or 16 bit integers and save in the corresponding file format.

(xi) Suppress noise in the reconstructed slices with the non-local means de-noising filter.

(xii) Generate orthogonal slices with vertical stitching if required.

An image viewer has been integrated into the *tofu ez* in order to facilitate the visual inspection of the CT slices and to clip the histogram of the reconstructed values. The advanced tab provides access to less frequently used algorithms and exposes performance optimization parameters. The fourth tab contains a number of tools for the stitching of images in case (1) the sample is larger than the beam and several local CT data sets were acquired to examine the entire volume; (2) for the preparation of the ‘half acquisition reconstruction’ (in parallel beam geometry, if a sample is rotated in the 0–360° range, two complete CT data sets are essentially acquired over the first and the second halves of rotation; if the rotation axis is shifted to the edge of the detector, the field of view can be effectively doubled).

### 3.3. Command line interfaces

The generic CLI program *ufo-launch* comes with *ufo-core* and not *tofu*, but we will shortly describe it for completeness. A workflow can be created by connecting multiple algorithms with exclamation mark, similar to chaining commands on the Linux command line using the pipe symbol. For instance, the following command reads images from a disk, bins and flips them in the left-right fashion and writes the results to the disk:[Chem scheme1] 
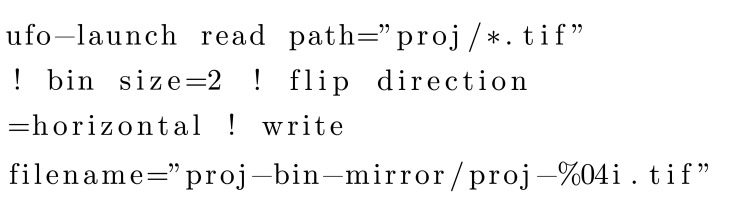


CLI-based sub-commands in *tofu* (see Table 2 ▸) contain predefined, parametrizable image processing flows. For instance, the following command performs flat field correction, fixes possible extreme values, computes the absorptivity and performs parallel beam tomographic reconstruction with the rotation axis in pixel 951, returning 200 slices around the vertical projection center with a spacing of 1 row:[Chem scheme2] 
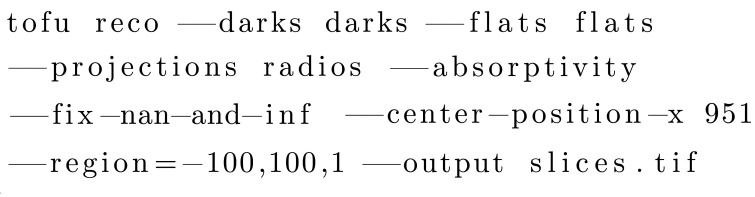


The following command performs phase retrieval based on the transport-of-intensity approach:[Chem scheme3] 
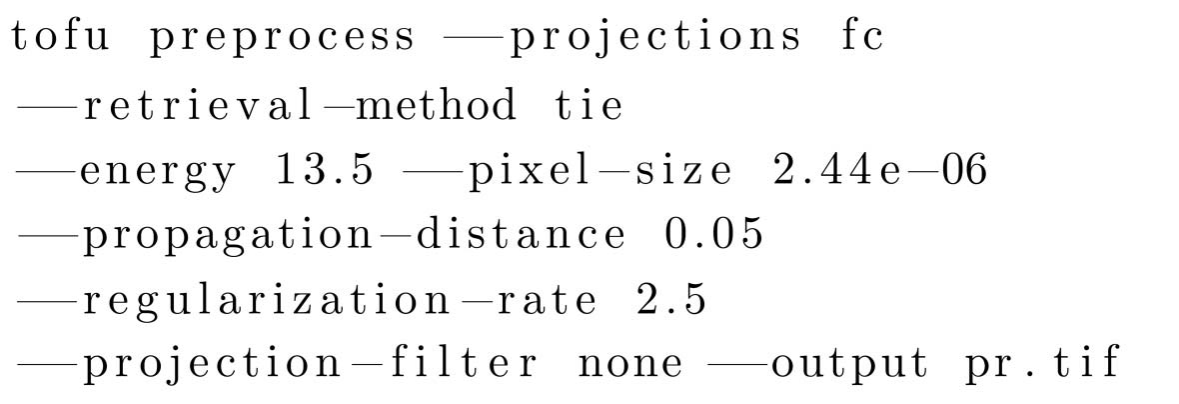


Experienced users can write scripts of any complexity or integrate workflows into custom software by using *ufo-launch* or one of the *tofu* CLI sub-commands. Moreover, they can use *tofu* as a library and use the workflows in their Python programs.

## 4. Application showcases

In the following sections we demonstrate the ability of *tofu* to reconstruct data of various geometries, X-ray setups and one from a neutron source. There will be four application showcases from various research fields with various imaging demands.

### 4.1. Parallel beam CT

**Figure 6:**
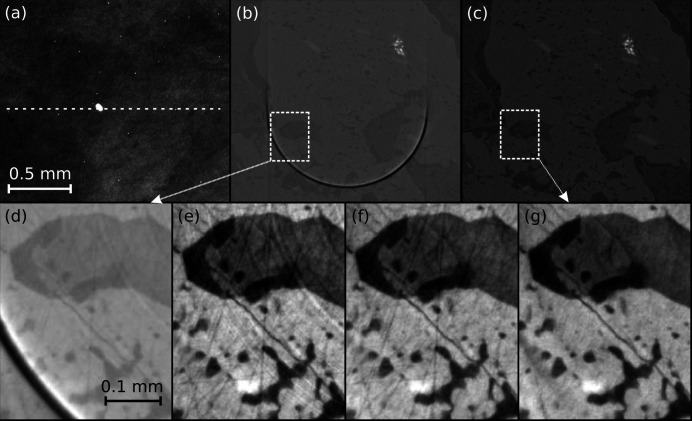
Reconstruction of parallel beam CT data with *tofu ez*. The sample is metamorphic schist (a piece of rock composed of four minerals). The top row shows from left to right fragments of: a raw CT projection (*a*) (dashed line indicating reconstructed row); of a slice reconstructed from phase-retrieved projections without the application of any artifact-reduction algorithms (*b*); of the same slice reconstructed with suppression of artifacts (*c*). Magnified fragments of images (*b*) and (*c*) are shown in insets (*d*) and (*g*), respectively. Images in the bottom row demonstrate progressive improvement when: only phase-retrieval was applied to data (*d*); phase-retrieval and broad ring removal (*e*); phase-retrieval, broad and narrow ring removal (*f*); all previous algorithms were applied and the outliers were removed from projections and flat-field images (*g*). Panels (*a*) and (*d*)–(*g*) have been inserted after automatic contrast adjustment was applied in *ImageJ* once to the entire image; no image correction of any kind was applied to panels (*b*, *c*). The scalebar for all images in a row is the same as shown in the heading image of that row.

In this subsection, we demonstrate the capability of *tofu* to provide a high-quality 3D reconstruction of a parallel beam µCT data set acquired at a synchrotron facility, in this case the Biomedical Imaging beamline of the Canadian Light Source. In addition to the 3D reconstruction, the data processing workflow included phase retrieval and various artifact reduction algorithms. The sample was a metamorphic schist mineral held in collections of the University of Calgary; see Fig. 6 ▸ for its projection and a slice with various pre-processing steps.

For the data acquisition, we used monochromatic beam with energy of 45 keV, an effective pixel size of 1.6 µm × 1.6 µm was obtained with a PCO Edge 5.5 camera coupled to a 50 µm LuAG:Ce scintillator (Crytur) by means of an optical system (Optique Peter) with 4× magnification. The distance between the sample and the detector was set to 20 cm. Approximately one-fifth of the camera dynamic range was used and 2000 projections were acquired. A fragment of the projection is shown in Fig. 4 ▸(*a*). One can notice a very large spot in the center and multiple bright pixels all over the image. The former is a defect typical for single-crystal scintillators, the latter occurs when a camera sensor gets hit by X-rays directly.

A CT slice from phase-retrieved projections without any additional processing [Fig. 6 ▸(*b*)] reconstructed at the detector row spoiled by the large spot exhibits a very intense ring artifact. It dominates the contrast and makes it almost impossible to interpret the image. Fig. 6 ▸(*c*) shows the same slice with various noise and artifacts suppression algorithms applied. Insets (*d*)–(*g*) in Fig. 6 ▸ show a fragment of the slice in order to highlight the degree of improvement as more algorithms were applied before reconstruction (the automatic contrast adjustment feature of *ImageJ* was used to improve the visual presentation). In the final slice and its fragment shown in Figs. 6 ▸(*c*) and 6(*g*), all artifacts are suppressed so that an accurate segmentation of four distinct minerals composing the specimen becomes possible (the bright white being ilmenite and the darkest, almost black mineral is quartz as confirmed by electron probe microanalyser).

### 4.2. Correlative neutron and X-ray CT

This subsection demonstrates the capability of *tofu* to provide 3D reconstructions of data from various probes, sources and imaging geometries. The experiment consisted of correlative dual-mode parallel-beam neutron and cone-beam X-ray tomography, conducted at the NeXT instrument (Tengattini *et al.*, 2020 ▸) at the Institut Laue Langevin in Grenoble (France). It is one of the few instruments world-wide with an additional cone beam X-ray microfocus CT setup installed to allow one to use complementary (attenuation) contrast provided by the two probes. At NeXT, neutrons come from a cold source inside the reactor enclosure and are transported via a curved guide to the instrument where, at the sample position, a maximum continuous flux of ∼3 × 10^8^ neutrons cm^−2^ s^−1^ is available.

**Figure 7:**
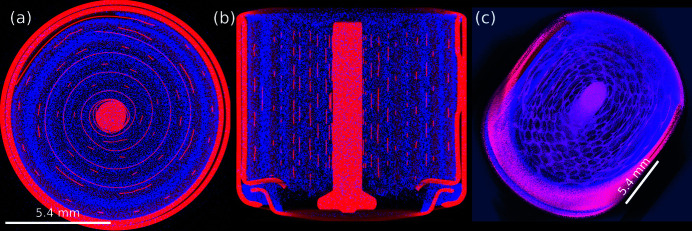
Combined neutron and X-ray tomography of a primary cell with LiMnO_2_ chemistry shown as a composite image. The blue channel depicts the linear neutron attenuation coefficient, the red channel the X-ray counterpart. Slices through the reconstructed volume along different directions are given in (*a*) and (*b*); a 3D rendering in (*c*). Cell diameter is 11.6 mm, voxel size 14.2 µm × 14.2 µm × 14.2 µm.

The sample was a lithium primary battery of type ‘CR1/3N’ which uses a LiMnO_2_ chemistry with an MnO_2_ cathode material. There is a genuine interest to investigate the different processes and phenomena during discharge of such batteries (and also during and after charging cycles of rechargeable batteries where aging processes are limiting their service lifetime) for different cell chemistries (Ziesche *et al.*, 2020*a* ▸,*b* ▸). Various slices and a rendering of the 3D volume combined from both probes is shown in Fig. 7 ▸.

Neutron tomography and laminography are based on the same principles as their X-ray counterparts. Here, neutrons serve as a probe to provide information about the local attenuation of a specimen in projection images acquired from different viewing angles. Since the neutrons activate the specimen and surrounding materials (like sample environments, sample manipulator parts or the detector), radioactive decay occurs and produces secondary particles and X-ray and gamma quanta. When gamma radiation hits the detector (either the scintillator of an indirect detector system or its light-sensitive sensor chip), white spots may appear on the 2D projection images. After 3D reconstruction, these spots are visible as streak or ‘zinger’ artifacts stretching across the slice’s planes, similar to the case described before for hard X-ray imaging. These artifacts can be efficiently reduced in *tofu* by filtering out the high-intensity spots in the projection images.

The indirect neutron detector was composed of a 10 µm-thick terbium-doped gadolinium oxysulfide (Gd_2_O_2_S:Tb) which was optically coupled (with slightly below 1:1 magnification) to a scientific CMOS camera (Hamamatsu ORCA-Flash4.0V2). Natively this camera has an array of 2048 × 2048 pixels of 6.5 µm pixel pitch which we used in 2 × 2-binning mode with an effective pixel size of 14.2 µm × 14.2 µm. We acquired 1600 neutron projections with 1 s exposure time in a scanning time well below 30 min which allows for time-resolved experiments during battery charging/discharging cycles.

The sealed microfocus X-ray tube at the NeXT instrument with a tungsten target and a beryllium window (Hamamatsu L12161-07) was operated at 120 kV acceleration voltage with a target power of 9.6 W. We acquired 1600 X-ray projections with an amorphous-silicon-based flat-panel detector with CsI scintillator (Varex PaxScan 2530HE) and 139 µm pixel pitch. The distance between the source and the sample was 38 mm and the distance between sample and detector 462 mm, resulting in a 13.2× magnification and effective pixel size 10.5 µm × 10.5 µm.

In Fig. 7 ▸, one can clearly see the complementary contrast obtained by the two probes. Neutrons are most sensitive to hydrogen and lithium (bright blue in the images), X-rays are mostly attenuated by heavier elements like copper, nickel and manganese, as well as the steel casing (bright red). A magenta cast occurs at locations where both X-ray and neutron attenuation is rather high, *e.g.* the MnO_2_ cathode material.

### 4.3. Helical cone beam CT

**Figure 8:**
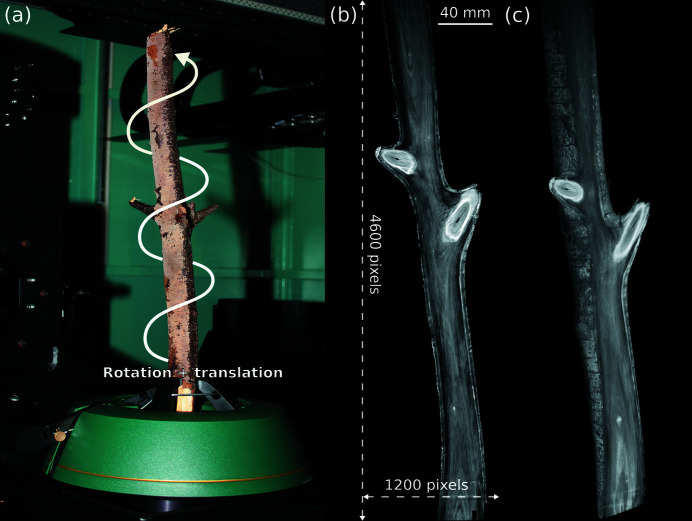
Helical cone beam tomography of a tree branch. Experimental setup (*a*); a slice through the reconstructed volume (*b*); 3D volume rendering (*c*). Total volume size was 2048 × 2048 × 5217 with voxel size 82.35 µm × 82.35 µm × 82.35 µm.

This example demonstrates the ability of *tofu* to reconstruct 3D data from complex geometries. In this case, helical cone beam CT was realized by simultaneous rotation of the sample and vertical translation of the X-ray source and the detector at the IPS X-ray imaging CL/CT-Laboratory. The sample was a tree branch, shown in Fig. 8 ▸ together with a reconstructed slice and a 3D rendering. The reconstruction was made possible by the ability to specify the vertical source and detector positions projection-wise.

An XWT-225 microfocus X-ray tube (X-RAY WorX) with a tungsten target, operated at an acceleration voltage of 160 kV with a target power of 75 W, was employed. The projection images were recorded with an XRD1612 flat-panel detector (PerkinElmer), featuring a physical pixel size of 200 µm × 200 µm with 2048 × 2048 pixels and a DRZ+ scintillator.

The distance between the source and the sample was 700 mm and the distance between sample and detector 1000 mm, resulting in a 2.4× magnification and effective pixel size 82.35 µm × 82.35 µm. We acquired 7265 projections in total (each exposed for 1.5 s), 2048 projections and 15 cm vertical shift per 360°. Thus, the fully reconstructable field of view was horizontally 168.6 mm and vertically 429.6 mm and the volume size was 2048 × 2048 × 5217 pixels.

### 4.4. Cone beam laminography

**Figure 9:**
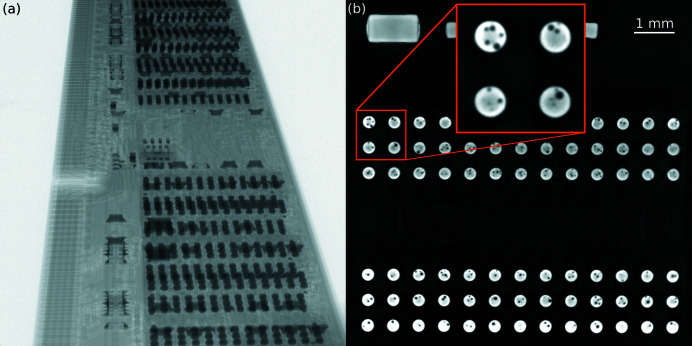
Flip-chip solder bump defects investigation in a DDR3 memory module by cone beam laminography. Projection image (*a*); reconstructed absorption slice (*b*) with a zoomed region showing some of the discovered defects.

This example demonstrates the ability of *tofu* to reconstruct cone beam laminography data. The measurement took place at the IPS X-ray imaging CL/CT-Laboratory. The sample was a conventional DDR3 memory module with flip-chip solder bump bonds. A projection from the data set together with a reconstructed slice which shows defects (voids) in the flip-chip solder bumps is shown in Fig. 9 ▸.

An XWT-225 microfocus X-ray tube (X-RAY WorX) with a tungsten target, operated at an acceleration voltage of 200 kV with a target power of 20 W, was employed. The projection images were recorded with an XRD1612 flat-panel detector (PerkinElmer), featuring a physical pixel size of 200 µm × 200 µm with 2048 × 2048 pixels and a DRZ+ scintillator.

The distance between the source and the sample was 85.5 mm, and the distance between sample and detector 1629 mm, resulting in a 20× magnification and effective pixel size 10 µm × 10 µm. The axis of rotation was inclined by 20.91° with respect to the tomography case. In total 2048 projections were taken over an angular range of 360° and every projection was exposed for four seconds.

## 5. Performance

**Figure 10:**
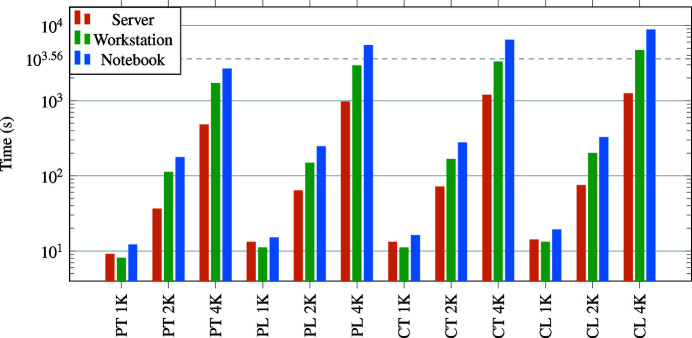
Filtered back projection compute times required by *tofu* on different systems, data set sizes (1K = 1024, 2K = 2048, 4K = 4096) and geometries (P = parallel, C = cone, T = tomography, L = laminography). One hour mark is shown as a dashed horizontal line.

With *tofu*, the overall throughput of systems with two or more GPUs can easily become limited by disk I/O performance instead of 3D reconstruction. Fig. 10 ▸ provides the reconstruction performance for different types of geometries and systems. A performance comparison with respect to some other reconstruction software is shown in Table 3 ▸. Throughout this section, the term *data set size* defines both the input and output sizes, *i.e.* for data set size *N*, the input are *N* projections of size *N* ^2^ and the output is a volume of size *N* ^3^.

The aim of the first benchmark was to show how fast our filtered back projection implementation is for different input sizes and reconstruction geometries on various systems. We used parallel beam tomography, parallel beam laminography, cone beam tomography and cone beam laminography geometries and data set sizes 1024, 2048 and 4096. Reconstruction performance was measured on a notebook with an Intel i9-10885H processor, 32 GB of RAM and an NVIDIA RTX 2070 Super graphics card with 8 GB of RAM. The second system was a workstation with Intel i7-7820X processor, 32 GB of RAM and two NVIDIA RTX 4000 graphics cards, each with 8 GB RAM. The most powerful system was a server with two Intel Xeon Silver 4114 processors, 256 GB RAM and four GeForce RTX 2080 Ti graphics cards, each with 11 GB RAM. We measured the wall time of *tofu reco* from invocation to return and used the so-called dry-run mode, where no data is read or written to the disk. Only the creation of empty OpenCL buffers, filtered back projection and downloading of the reconstructed volume from graphics memory to main memory was performed. In this, as well as in the second benchmark below, no flat field correction or other pre- and post-processing steps were applied and the input and ouput data were in single-precision floating-point arithmetic. Fig. 10 ▸ summarizes the performance results.

The second benchmark is summarized in Table 3 ▸ and compares the performance of *tofu*, *ASTRA* (van Aarle *et al.*, 2016 ▸) and *PyHST2* (Mirone *et al.*, 2014 ▸). We again measured the wall time and this time also included the disk I/O in order to provide a more realistic estimate of real-world reconstruction times. The disk was a RAID 0 built from two Samsung 860 EVO 500 GB SSDs with a final throughput of about 1.3 GB s^−1^. We used parallel beam tomographic geometry. All benchmarks were performed by using all four GPUs of the server described above and in all packages we used the filtered back projection algorithm. Opposed to slice-by-slice reconstruction, *ASTRA* does not support filtering in the volume-based reconstruction, so in that case we performed only the back projection part. Similar benchmarks on a small CPU-based cluster can be found in Marone *et al.* (2017 ▸).

Tomographic image processing can be massively parallelized and is thus extremely well suited for GPU implementation, which is true also for additional frequency filtering steps. For example, flat field correction and filtered back projection of a data set with size 2048 on the workstation mentioned above took *tofu* 143 s including reading from file and writing of results to disk. When we added phase retrieval based on the transport-of-intensity approach to the workflow, the processing took 159 s, which increased the total reconstruction time by only 11%.

## 6. Conclusion

We presented *tofu*, a set of versatile high-level user interfaces for tomographic image processing including a command-line interface for efficient scripting and two graphical user interfaces for user-friendliness. It includes many pre- and post-processing algorithms, such as phase retrieval and ring-removal and supports parallel beam, cone beam, tomographic and laminographic geometries. Thus it can reconstruct data acquired with different types of light sources, setups and complicated geometries, as we have demonstrated by several use cases. The modularity makes this an ideal tool for method development platforms, where data processing workflows need to be easily adjustable.

The image processing code can run on GPUs, therefore the package is not only user-friendly but also fast and, as the performance measurements show, users can obtain reconstructions in short times even by using ordinary, off-the-shelf hardware. The combination of a GUI and fast processing permits one to work with data in an interactive way, which is very useful for fine-tuning algorithm parameters and teaching people how to perform tomographic reconstruction. Once all parameters are optimized, multiple data sets acquired under the same experimental conditions can be sent to batch processing.

The presented software has been used at KARA, CLS and ESRF synchrotrons and ILL neutron source for many years and received a lot of positive feedback from users. We believe that the variety of implemented algorithms, speed and cost-efficiency of GPU computing combined with simple graphical user interfaces will make the presented toolkit very attractive to a broad community of synchrotron and neutron users and researchers who need software for their custom-build laboratory-based microtomography systems.

## Figures and Tables

**Table 1:** *Tofu* software stack

Component	Description
*tofu*	Python-based library of CLIs and GUIs for user-friendly creation of image processing workflows (https://github.com/ufo-kit/tofu, https://tofu.readthedocs.io)
*ufo-filters*	Library of image processing algorithm implementations, including tomographic reconstruction (https://github.com/ufo-kit/ufo-filters, https://ufo-filters.readthedocs.io)
*ufo-core*	GPU-enabled execution of image processing workflows on multi-GPU systems (https://github.com/ufo-kit/ufo-core, https://ufo-core.readthedocs.io)

**Table 2:** User interfaces and their typical use cases

Program	UI type	Use case
*tofu flow*	GUI	Visual workflow programming
*tofu ez*	GUI	Batch-processing of many tomographic data sets
*tofu preprocess*	CLI	Pre-processing workflows including phase retrieval
*tofu find-large-spots*	CLI	Finding spots of extreme intensity
*tofu sinos*	CLI	Sinogram generation
*tofu tomo*	CLI	Parallel beam tomographic reconstruction from sinograms
*tofu reco*	CLI	Cone/parallel tomographic/laminographic reconstruction from projections
*ufo-launch*	CLI	Creation of arbitrary workflows on the command line

**Table 3:** Reconstruction times of different software packages for different data set sizes The results are in form mean (standard deviation) computed from ten runs. Values in bold indicate the fastest package for a given data set size.

	Data set size		
Package	1024	2048	4096
UFO	**12** (0.15) s	**74** (0.89) s	947 (12.84) s
PyHST	18 (0.12) s	93 (0.89) s	**770** (9.39) s
ASTRA	25 (1.39) s	189 (5.93) s	1777 (42.99) s
